# Multiple-pollutant cost-efficiency: Coherent water and climate policy for agriculture

**DOI:** 10.1007/s13280-019-01257-z

**Published:** 2019-09-24

**Authors:** Sanna Lötjönen, Markku Ollikainen

**Affiliations:** Department of Economics and Management, University of Helsinki, P.O. Box 27, 00014 Helsinki, Finland

**Keywords:** Agriculture, Greenhouse gas emissions, Marginal abatement cost, Multiple pollutants, Nutrient runoff

## Abstract

We examine the abatement costs for water and climate pollutants and their respective policies while accounting for cobenefits. We construct private and social marginal cost curves for reducing greenhouse gas emissions and nutrient runoff in Finnish agriculture. We find that the social marginal costs of reducing emissions that reflect the cobenefits are lower than the private costs. Accounting for greenhouse gas cobenefits from nutrient load reduction or water cobenefits from climate emissions reduction creates a gap between privately and socially optimal reduction levels. This gap varies depending on the valuation of cobenefits. The cost-efficient reduction of the focus pollutant is increased when cobenefits from the other pollutant are accounted for. For policies, this implies a higher cap or tax on the focus pollutant. We decompose the optimal tax rate to a basic tax on the focus pollutant and on an additional tax component depending on the level of cobenefits.

**Electronic supplementary material:**

The online version of this article (10.1007/s13280-019-01257-z) contains supplementary material, which is available to authorized users.

## Introduction

Agriculture contributed approximately 10–12 % of the anthropogenic CO_2_-equivalent emissions worldwide in 2010 (IPCC 2014). This is an alarmingly high fraction that indicates the need for reductions in agricultural greenhouse gas (GHG) emissions. Further, agriculture has a considerable impact on local and regional water quality due to nutrient loading. In many areas, such as the Baltic Sea region, agriculture contributes approximately 50 % of the regional nutrient loading (HELCOM 2018). The use of nitrogen especially impacts both water and airborne emissions from agriculture. Thus, any change in nitrogen fertilization will change the nutrient loading and GHG emissions. The same interdependence holds true, for instance, for changes in land allocation between crops. In many cases, water policy creates climate benefits as cobenefits, and vice versa; however, policies sometimes promote measures that work against each other.

A generally accepted principle in environmental policy is that of cost-efficiency. In its simplest form, it requires policy that ensures that the marginal costs (MCs) for emission reductions of all polluters are equal. This principle is simply a solution to an abatement cost minimization problem that is subject to a given emission reduction target. Marginal abatement costs reflect the properties of the abatement cost functions and define the cost of abating one additional unit of emissions. Marginal cost curves (MCCs) are convenient for policy-makers, as they provide the possibility to compare abatement costs not only within a sector but also between different sectors, such as point sources and nonpoint sources in water policies, or abatement costs in the national transport sector and carbon prices in the EU Emissions Trading Scheme in climate policies.

MCCs can be derived using different approaches, including a bottom-up approach, with a supply-side, a microeconomic and/or engineering model, or a top-down approach, with equilibrium models (e.g., Vermont and De Cara 2010). Each approach has its advantages and disadvantages (e.g., Eory et al. 2018). Here, we use a bottom-up approach to derive the costs of emissions reductions because it best reflects the fundamental production conditions, technological possibilities and constraints in agriculture. We want to assess how the costs of emissions reductions behave when cobenefits are taken into account. Intuitively, cobenefits reduce the costs of any policy; however, it is interesting to ask how much of an effect cobenefits have and what policy conclusions can be drawn from their presence. In this paper, we specifically assess how much the costs of water policies are reduced when climate benefits are taken into account and how much climate policy costs are reduced when water quality benefits are taken into account. Additionally, we examine how the costs of reducing nutrients and GHG emissions relate to those in other sectors.

Despite the importance of the simultaneous analysis of measures targeting multiple pollutants, rigorous studies are relatively scarce (see for example Brink et al. 2005; Schneider et al. 2007; MacLeod et al. 2010; Ambec and Coria 2013; Eory et al. 2013). As a recent review by Eory et al. (2018) emphasizes, it is of utmost importance to account for negative and positive coeffects of agricultural mitigation practices. Our paper builds on this importance.

A number of studies have calculated or reviewed MCs for reducing GHG emissions; for example, Dequiedt and Moran (2015) used legumes in French agriculture; O’Brien et al. (2014) compared IPCC (Intergovernmental Panel on Climate Change) guidelines and life-cycle analysis for Irish agriculture; MacLeod et al. (2010) and Moran et al. (2011) for UK agriculture; De Cara and Jayet (2011) for European agriculture; Beach et al. (2008) for 36 world agricultural regions; Bosello et al. (2007) for European countries with an emphasis on policies, and Eory et al. (2018) reviewed MCCs for European agriculture with an engineering approach. None of these papers include the abatement costs of nutrients and water policies.

Separate studies that calculated or reviewed agricultural MCs for nutrient load reductions include, for example, Helin et al. (2006) for crop production and Helin (2014) for dairy management in Finland. Elofsson (2003) provided the cost of reducing nutrient loading for the Baltic Sea region but did not include GHG emissions. Rigorous analyses of the costs of reducing GHG emissions in Finnish agriculture are lacking. We derive MCCs with cobenefits for Finnish agriculture by focusing on GHG emissions and nutrient runoff and on the cobenefits from the abatement measures. To our knowledge, this is the first attempt to examine the interaction between the two types of pollutants in the Baltic Sea region.

## Methods: Constructing cost functions for emissions reductions

In this section, we first present how to derive cost functions and marginal cost functions for reducing emissions of one pollutant. Then, we introduce a method to account for multiple pollutants as cobenefits from abatement measures.

Cost functions define the minimum real costs of producing a certain outcome; in our case, the minimum cost of abating water and climate pollutants. Abatement cost functions are derived in relation to a chosen baseline, typically relative to private profits that are optimized in the absence of any reduction requirements to emissions. Imposing gradually tightening limits on emissions and letting the agent take required measures to meet the target produces the following private abatement cost for each measure *m* at each level of emissions:

1 $$ C_{s}^{m} = \frac{{{\text{Private real costs}}_{s}^{m} }}{{{\text{Emissions reduced}}_{s}^{m} }} , $$

where *s *= *i, j* denotes either GHG emissions or nutrient loads. The cost function for one mitigation measure is derived by fitting a function to the separate cost levels as a function of the intensity of the measure. Finally, these cost functions can be aggregated to obtain a more general cost function for multiple measures of agricultural abatement.

Equation (1) represents the case when only one pollutant is considered. We are ultimately also interested in how mitigation measures directed to nutrient loads or GHG emissions affect other pollutants and further the social abatement cost of these measures. We calculate the social cost of reducing emissions of two pollutants by following the method of Eory et al. (2013). The social reduction cost comprises the private real cost and the reduced or increased damage cost of the other pollutant that results from considering any given mitigation measure. Adding this external cost to the private cost can thus increase or decrease the total social cost of emission reductions. The social cost SC is determined for each mitigation measure $m$ and pollutant $i$ as follows (Eory et al. 2013; 57).

2 $$ {\text{SC}}_{i}^{m} = \frac{{{\text{Private real costs}}_{i}^{m} + \left( {{\text{Change in emissions}}_{j}^{m} \times {\text{Damage cost}}_{j} } \right)}}{{{\text{Emission reduced}}_{i}^{m}  }} $$

To separate the cost functions in Eqs. (1) and (2), we define the first equation without cobenefits as a private cost function and the second with cobenefits as a social cost function. These definitions are used throughout the article.

Equations (1) and (2) represent the costs when mitigation measures are implemented separately. Aggregating over all measures yields the aggregated total cost function of emission reductions for agricultural pollutants. Once these costs functions are known, societies minimize the sum of abatement costs for a given target of emissions reduction. *E* denotes the emissions reduction target, which is lower than the prevailing emissions. $C_{i} \left( {q_{i} } \right)$ is the private cost function, where $q$ denotes the emission reductions of a polluter *i* (*i* = 1,…,n). Then, the solution to this constrained minimization problem ($\hbox{min} \sum\nolimits_{i = 1}^{n} {C_{i} \left( {q_{i} } \right)}$ subject to $\sum\nolimits_{i = 1}^{n} {q_{i} \ge E}$) gives the cost-efficiency condition for all polluters *i* and *j*:

3 $$ {\text{MC}}_{i} \left( {q_{i}^{*} } \right) = {\text{MC}}_{j} \left( {q_{j}^{*} } \right) , $$

where MCs refer mathematically to the derivative of the cost functions of emissions reduction. Condition (3) simply requires that emissions reductions are allocated to polluters in such a way that their marginal costs are equal. We next develop the marginal costs of emissions reduction for our case.

## Deriving marginal costs of emissions reductions

The required steps for developing MCCs are presented, for example, in Moran et al. (2011). We next present the core assumptions used in our analysis and the chosen mitigation measures.

### Assumptions

For the calculation of abatement costs and potentials, we use a bottom-up approach. As alternative baselines for calculating reductions in GHG emissions and nutrient runoff, we employ (1) a private profit maximization under the free market (no policies to reduce emissions in place), and (2) a private profit maximization under agro-environmental policies (for the year 2018; including agricultural area-based subsidies). We use a discount rate of 3 %. Mitigation measures are presented in the next section. For the abatement costs and potentials, we consider average values for Finland, separated for mineral and organic soils. We focus on carbon dioxide (CO_2_), nitrous oxide (N_2_O) and methane (CH_4_) emissions as CO_2_-equivalents (CO_2_e) and nitrogen (N) and phosphorus (P) loads (including particulate phosphorus (PP) and dissolved reactive phosphorus (DRP)) as nitrogen equivalents (Ne).

Cost functions are derived by placing limits on GHG emissions and nutrient runoff compared to the baseline values, ranging from 0 to 50 %, and by optimizing private profits with the given constraint. Aggregation of cost functions from different measures is achieved by minimizing the total costs of emissions reductions while gradually increasing the required abatement. For aggregation, we estimate the total applicable hectares for each measure (see ESM 2 for details). For some measures such as afforestation, we obtain a specific emissions reduction with a specific abatement cost, and for such measures, cost functions cannot be derived. We also account for possible no-regret situations, i.e., win–win solutions, where abatement costs are negative.

### Mitigation measures considered

We focus on a limited set of measures and provide our analysis on emission reduction measures separately for crop production and dairy management. The measures are selected based on their estimated efficiency, feasibility, and data availability. Table 1 shows the studied measures, as well as the affected GHG emissions and nutrients in runoff. We indicate the impacts as increasing (+), decreasing (−) or absent (0). Superscript a accompanying some variables indicates that the value of the variable has changed to reduce emissions and loads (decreased for herd size and fertilization and increased for buffer strips).

**Table 1:** Studied measures to reduce GHG emissions and nutrient runoff, the affected pollutants by each measure and the direction of change for each pollutant

	CO_2_	N_2_O	CH_4_	N	PP	DRP
Dairy management						
Herd size^a^	±	±	−	−	−	−
Diet (share of concentrates)^a^	0	0	±	0	0	0
Fertilization (mineral/manure; amount)^a^	0	−	0	−	−	−
Exporting manure^a^	0	0	0	−	−	−
Land allocation (silage/cereal)^b^	−	0	0	−	−	−
Manure storage (without cover/floating cover)^b^	0	−	+	0	0	0
Manure spreading (injection/broadcast)^b^	0	−	0	0	−	−
Crop production						
Fertilization (amount)^a^	−	−	0	−	−	−
Buffer strips (width)^a^	−	0	0	−	−	−
Legumes in crop rotations	−	−	0	−	0	0
Catch crops	−	−	0	−	0	0
Tillage method (conventional/no-till)^b^	−	−	−	−	−	−
Afforestation	−	+	−	−	−	−
Green fallow	−	+	−	−	−	−

Based on Lötjönen et al. (unpubl. results), Ervola et al. (2012, 2018), Valkama et al. (2015), Lötjönen and Ollikainen (2017)

+ GHG/nutrients increased, − GHG/nutrients decreased, 0 GHG/nutrients are not affected by the measure

*CO*_2_ carbon dioxide, *N*_2_*O* nitrous oxide, *CH*_4_ methane, *N* nitrogen, *PP* particulate phosphorus, *DRP* dissolved reactive phosphorus

^a^Continuous measure; the other measures are considered “technological choices”, i.e., either applied or not; the level of the measure is assumed to decrease or increase to reduce emissions and loads when determining the direction of change in GHG/nutrients (increasing for exporting manure and buffer strips, decreasing otherwise)

^b^The first option in parentheses is compared to the second option when determining the direction of change in GHG/nutrients

The derivation of abatement cost functions for dairy farms is based on the dairy management model by Lötjönen et al. (unpubl. results). The authors studied an average farm with both milk production and crop cultivation. The farmer maximized profits from milk production by choosing the herd size, diet (shares of silage and concentrate feed), manure storage coverage (uncovered or floating cover), manure spreading method (broadcast or injection), number of milking seasons, land allocation between barley or silage and fertilization (manure or mineral fertilizer). All individual measures were solved simultaneously in the model; thus, interrelations between measures were endogenously taken into account. Measures for dairy management were applied only to mineral soils due to insufficient information.

When deriving the abatement cost functions for crop production, barley is used as a representative cereal crop. Continuous measures to reduce GHG emissions and nutrient runoff include decreasing mineral fertilization and increasing buffer strips. As discrete technological choices, the decision-maker may choose between conventional tillage or no-till and whether to apply crop rotation with legumes, afforestation, green fallowing, or catch crops. Calculations for legumes in crop rotations are based on Lötjönen and Ollikainen (2017), for catch crops they are based on Valkama et al. (2015), and for other measures they are based on Ervola et al. (2012, 2018). Most measures in crop production are applied to both mineral and organic soils, but crop rotations and catch crops are considered for mineral soils only.

As the baseline for reducing GHG emissions or nutrient runoff, we use the private optimum under either free market or Finnish agro-environmental policy in 2018 (CAP). Social coeffects from nutrient runoff are valued at 9 € kgNe^−1^, and those from GHG emissions are valued at 35 or 50 € tCO_2_e^−1^. Please see supplementary material (ESM 1) for a more detailed description of the mitigation measures considered.

## Results

In the following section, we present the main results using the free market as the baseline. Details of all results are allocated to the electronic supplementary material (ESM 2, with Tables S13 and S14 presenting the total cost functions). We first present the results for dairy management and then for crop production. Multiple pollutants are discussed within both production lines. As a final step, we examine the aggregate marginal cost curves and the implications of multiple pollutants for policy design.

### MCCs for dairy management

**Fig. 1:**
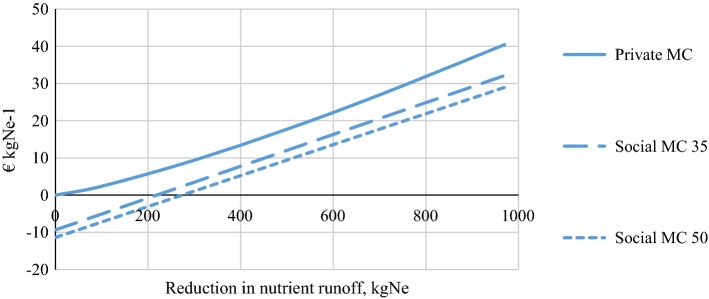
The private and social marginal costs of nutrient runoff reductions in dairy management

**Fig. 2:**
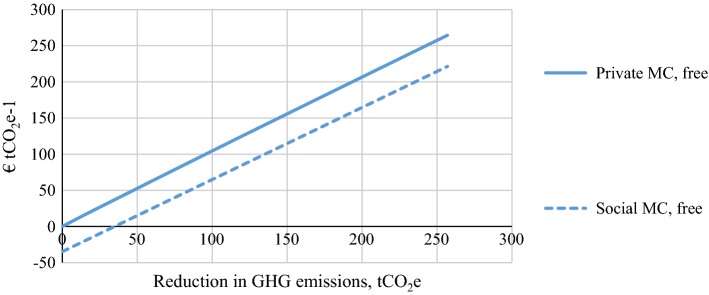
The private and social marginal costs of GHG emission reductions in dairy management

The MCCs of nutrient loading and GHG emissions reduction in dairy management are presented in Figs. 1 and 2 (Figs. S1 and S2 in ESM 2 include also the MCCs for the CAP baseline). They are based on the functions fitted to reductions in pollutants up to 50 % from the baseline level (3233 kgNe in Fig. 1 and 514 tCO_2_e in Fig. 2; see Table S3 in ESM 2 for details). The horizontal axis in all figures denotes either reduction in GHG emissions in kilograms or tons of CO_2_-equivalents (CO_2_e) or reduction in nutrient runoff in kilograms of N-equivalents (Ne).

In the free market baseline, the dairy farm has 61 dairy cows with three milking seasons, floating cover for manure storage, manure broadcast spreading, no manure exports, four out of ten field parcels in barley cultivation and concentrate intake of 17.5 kg DM day^−1^, with total GHG emissions of 514 tCO_2_e farm-1 and total nutrient runoff of 3233 kgNe farm-1 (see details in Lötjönen et al. unpubl. results, and in ESM 1 and 2). The levels of individual measures for each abatement level and for both baselines are presented in the supplementary material (Table S2 in ESM 2).

Marginal costs of reducing nutrient runoff are presented in Fig. 1. We use euros per unit of reduction to facilitate an easy comparison with the respective values of crop production in the next section (the same applies for Fig. 2). The solid curve denotes the abatement costs in the absence of cobenefits (private marginal abatement cost) and the dashed lines include cobenefits from reduced GHG emissions valued by the social cost of carbon—either 35 € tCO_2_e^−1^ or 50 € tCO_2_e^−1^ (social marginal abatement cost). The difference between the solid and the dashed lines represents the reduction in climate damage as a cobenefit of the measures taken for nutrient runoff reductions and weighted by the nutrient load reduction. The private and social marginal cost curves are increasing and almost linear or slightly convex, reflecting strongly increasing total reduction costs.

Figure 1 indicates that for every reduction of nutrient runoff, the social marginal costs are lower than the private ones. The vertical distance between private and social cost curves indicates the savings in abatement costs of GHG emissions weighted by the reduced loading. For example, setting a tax equal to 10 € kgNe^−1^ would yield a reduction of approximately 300 kgNe in the private solution but more than 400 kgNe as the social solution when cobenefits are accounted for. This finding will be discussed further in later sections.

Figure 2 illustrates the marginal costs of reducing GHG emissions. The above analysis holds true for Fig. 2 as well. The private and social MCCs for GHG emissions are rather linear, reflecting increasing and convex total abatement costs. Interestingly, the first units of reduction provide negative social marginal costs after which they tend to increase, indicating that the first reduction units produce net benefits for society. Looking at the changes in individual measures (see Table S2 in ESM 2), we notice that GHGs and nutrient runoff are mainly reduced by decreasing herd size. Additionally, the share of concentrates, number of barley parcels and overall fertilization level (not shown) decrease as the GHG or runoff limits tighten.

### MCCs for crop production

When deriving MCCs for crop production, we account for measures that modify cultivation towards better environmental performance without shifting land to other purposes. These kind of measures are the cheapest in terms of euros per hectare, as profits from yields are not lost even though they can be lower. Here, GHG emissions or nutrient runoff are reduced by decreasing mineral fertilization and increasing the share of buffer strips (the levels of mineral fertilization and buffer strips for each measure and abatement are presented in Tables S4–S7 in ESM 2). As previously stated, nutrient runoff is expressed in N-equivalents, so the effects of N and P runoff are not separated. Costs for green fallow and afforestation are presented in Tables S10 and S11 in ESM 2. It should be noted that in practice, afforestation changes the land use from agriculture to forestry, and the farmer loses the area-based subsidies. This provides great difficulties for a policy-promoting afforestation.

**Fig. 3:**
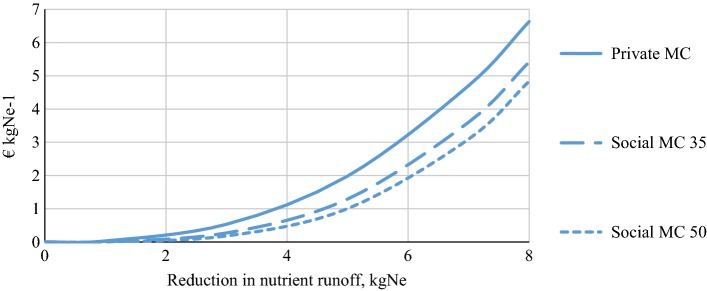
Aggregated marginal cost curves for nutrient runoff reductions in crop production with measures allowing for cultivation

**Fig. 4:**
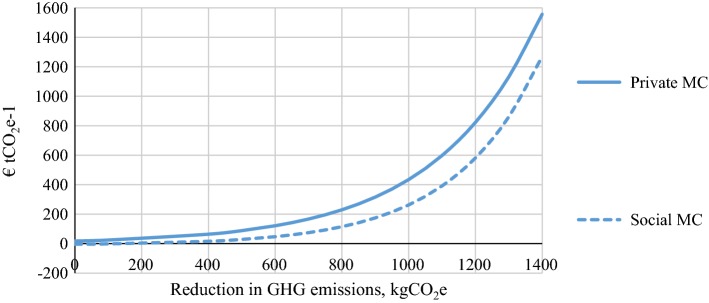
Aggregated marginal cost curves for GHG emission reductions in crop production with measures allowing for cultivation

We first calculated the marginal abatement costs of nutrient runoff (€ kgNe^−1^) and GHG emissions (€ tCO_2_e^−1^) separately for each measure (see Tables S3–S8 in ESM 2 for details). Then, we aggregated the cost functions by minimizing the total abatement costs while varying the required abatement amount and accounting for the estimated total applicable areas for each measure. Figure 3 illustrates the aggregated private and social marginal cost functions for reducing nutrient runoff. Similarly, Fig. 4 presents the marginal private and social cost curves for reducing GHG emissions (Figs. S9 and S10 in ESM 2 include also the aggregated MCCs for the CAP baseline). Tables S8 and S9 in ESM 2 present the abatement amounts for each measure under different total abatement levels. For reducing both nutrient runoff and GHG emissions, most of the reductions come from organic fields.

Comparing Figs. 1 and 3, the difference between private and social marginal costs of nutrient load reductions is larger in dairy management. However, Fig. 4, when compared with Fig. 2, suggests that the difference between private and social GHG marginal costs is greater in crop production than in dairy management. Therefore, reducing GHG emissions in crop production provides more cobenefits in the form of reduced nutrient runoff. It should be noted that the horizontal axis in Fig. 2 for dairy management is in tons of CO_2_e, while the horizontal axis in Fig. 4 for crop production is in kilograms of CO_2_e. Marginal costs of reducing GHG emissions are higher in crop production than in dairy management. In dairy management, the potential reductions in both pollutants are greater than those in crop production.

A measure that would shift land use to another purpose is, for example, afforestation. It provides nutrient runoff and GHG emission reductions, for which both private and social marginal costs are considerably lower (relative to the reduced amount of GHG) than costs indicated in Figs. 3 and 4. For example, reducing GHG emissions in organic soils would reduce 12.7 tCO_2_e ha^−1^, with a cost of 23.2 € tCO_2_e^−1^ (see ESM 2). It should be noted that here, emission reductions are hectare-based in contrast to Figs. 1 to 4. Green fallowing would be a costly measure for reducing nutrient runoff in mineral soils; however, green fallowing in organic soils provides an efficient option for reducing GHG emissions (9.9 tCO_2_e ha^−1^ reduced with a private marginal abatement cost of 29.5 € tCO_2_e^−1^ in CAP baseline). Even though afforestation is a cost-efficient measure for society in marginal costs, private per hectare costs would be high due to the lost area-based subsidies.

It should also be noted that the cost per hectare in afforestation is identical for nutrient runoff or GHG emissions reductions. This result demonstrates that ignoring or accounting for other pollutants or other effects may have a strong influence on which measures are preferred when ordered by cost-efficiency. In addition to GHG emissions and nutrient runoff, the measures could be evaluated based on their effect on biodiversity, soil carbon or landscape. The cost for reducing nutrient runoff or GHG emissions is generally lower under free market conditions than under CAP (see ESM 2), as the baseline pollution levels are higher (no regulation) and, thus, the required reduction in absolute numbers is also lower.

A direct comparison of marginal costs between crop production and dairy management without aggregating them is difficult because the former is expressed in per hectare terms and the latter in absolute terms for a given dairy farm size. To facilitate an illustrative comparison of the two production lines, the MCC for dairy management needs to be modified to a per hectare basis. It should be noted that this modification is arbitrary, as the field area of dairy farms may vary greatly, and a dairy farm might not even have any fields. For illustration purposes, we divided the private costs and GHG emissions reductions by 102 (i.e., the total field area of the modeled farm) and plotted this curve with the crop production MCC (please see Fig. S11 in ESM 2). Here, the marginal costs for dairy and crop production are similar, but the curve for dairy shifts depending on the field area. Thus, the relevant comparison can only be achieved by aggregating both curves together, which is performed in the next section.

### MCCs for the entire agricultural sector and policy designs

**Fig. 5:**
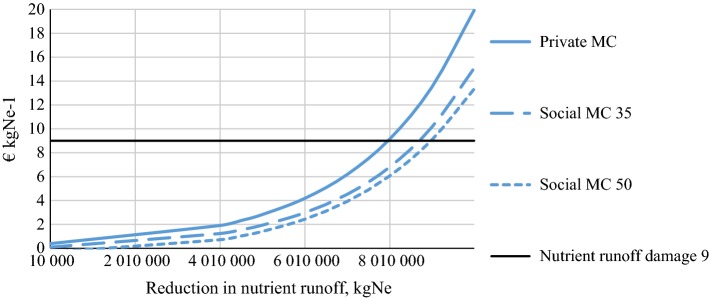
Marginal cost curves for nutrient runoff reductions in crop production and dairy management, combined

**Fig. 6:**
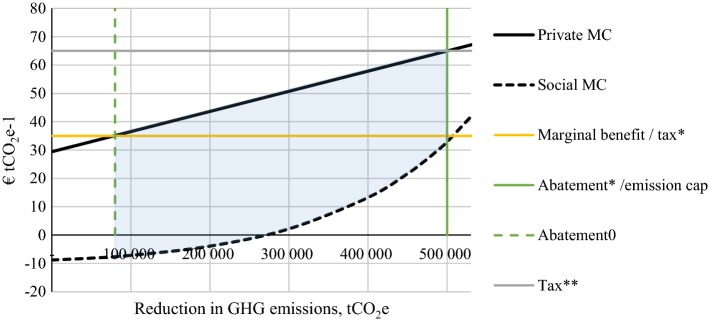
Marginal cost curves for GHG emissions reductions in crop production and dairy management, combined under a free market baseline, and an illustration of policy options

Finally, we aggregate private and social costs of emissions reductions over dairy management and crop production (Figs. 5 and 6; see Figs. S12 and S13 in ESM 2 for the CAP baseline) to assess the cost-efficient solution between agriculture and other sectors. For water policies, we compare agriculture and wastewater treatment plants, and for climate policy, we compare the cost in agriculture with alternative carbon prices.

Marginal costs of reducing nutrient runoff from sewage water in average-sized wastewater treatment plants (WWTPs) amount up to approximately 8 € kgN^−1^, with an abatement of up to approximately 200 tN compared to current loadings (Hautakangas et al. 2014). In our results, an abatement with the same cost would reach approximately 7700 tNe from crop and dairy management combined. Figure 5 presents an aggregated cost function, indicating that nutrient reductions result from a larger set of measures. This analysis reveals that when focused as a whole, agriculture has greater possibilities to reduce loads than solely considering the potential of field parcels. Table S12 in ESM 2 reveals that the largest share of nutrient runoff reductions stem from dairy management measures. We outline the policy when discussing Fig. 6. This discussion would fit this case as well.

Figure 6 represents MCCs for reducing GHG emissions; reductions in GHG emissions originate mainly from dairy management measures. In addition to MCCs, Fig. 6 includes carbon price, 35 € tCO_2_e^−1^, which, in this context, is also interpreted as the marginal benefit from GHG emission reductions. With this (constant) marginal benefit function, the privately optimal abatement level would be less than 100 000 tCO_2_e. This value falls short of the corresponding socially optimal abatement level, approximately 500 000 tCO_2_e. Including cobenefits from changes in nutrient loads creates a gap between the socially and privately optimal abatements.

A straightforward possibility for achieving the socially optimal abatement level would be to impose an emissions cap (abatement*/emission cap in Fig. 6). A market-based instrument could be an emissions tax, set at the level where the social marginal costs equal the marginal benefits. We can divide this solution into two stages. If the cobenefits are ignored, then the optimal abatement would be abatement 0, and the associated tax is the yellow line. Accounting for cobenefits shifts the MCC outwards, defining the optimal abatement* and a tax rate reflecting it. Theoretically, this tax** consists of two components: a tax rate that reflects the bare abatement costs of GHG emissions (tax*) and an incremental part (tax** − tax*) that reflects the water cobenefits. The blue-shaded area in Fig. 6 indicates a positive externality from climate policy on nutrient policy. Even though the optimal tax (tax**) provides the required solution, a lump sum subsidy, compensating for this externality, would be possible.

## Discussion

Our results show that the marginal costs for reducing both nutrient runoff and GHG emissions are higher in crop production than in dairy management. This differs slightly from previous studies, which find that marginal costs for livestock-related measures are approximately within the same range as crop production measures (see e.g. Eory et al. 2013). We also found that GHG emissions reduction in dairy management is accompanied by relatively high nutrient runoff cobenefits, which indicates the need to strengthen regulations on nutrient loads in dairy farms to establish the equality of marginal costs between these two production lines.

As for climate policies, crop production measures in this study would reduce emissions only slightly with a cost below an estimated value of the social cost of carbon (approximately 35 € tCO_2_^−1^; Tol 2011). The main reductions are obtained from organic soils with conventional tillage and fields in crop rotation. Also afforestation in organic soils, with a cost of 23.2 € tCO_2_e^−1^ under the free market, has a cost below that value. The level of marginal costs for wastewater in WWTPs suggests that for reducing nutrient runoff in agriculture under CAP, the abatement amounts are rather small and have comparably high marginal costs. This result is in line with the results of Hautakangas et al. (2014), which emphasize that there are still many possibilities to abate nutrients in WWTPs. Results using buffer zones to reduce nutrient loadings from clearcut forests in Finland suggest a marginal abatement cost of approximately 500 to 2500 € kgNe^−1^ for an 10 to 30% reduction (i.e., 0.85 to 2.55 kgNe ha^−1^) (Miettinen et al. 2019). These cost estimates for forests are considerably higher than our estimates for agriculture.

In crop production, the marginal costs derived for reducing nutrient runoff are comparable to the work of for example Helin et al. (2006), who found an average abatement cost of 7.2 € kgN^−1^ from aggregated reduction in southwest Finland in 2006. Our results from dairy management correspond quite well with Helin (2014), where the marginal cost for nitrogen varies between 7.0 and 24.8 € kgN^−1^ for the chosen abatement levels. De Cara and Jayet (2011) estimated a cost of 32 to 42 € tCO_2_e^−1^ for reducing GHG emissions from agriculture by 10% at the EU level. A similar magnitude is found in our cost estimates in the higher abatement levels.

Accounting for cobenefits provides an important twist to the results, as noted also by Eory et al. (2013) and Ervola et al. (2018). For example, in our results reducing GHG emissions by 15%, compared to the free market baseline in barley cultivated with conventional tillage in mineral soils (i.e., 372 kgCO_2_e ha^−1^), reduces nutrient runoff by 16 kgNe ha^−1^ as a cobenefit. In the same setting, reducing nutrient runoff by 15% (i.e., 4 kgNe ha^−1^) lowers GHG emissions by 21 kgCO_2_e ha^−1^. For the same reduction, valuing climate damage with 50 € tCO_2_e^−1^ yields a higher GHG cobenefit than with a value of 35 € tCO_2_e^−1^. The reduction levels and the used damage values then in turn affected the social MCs of emission reductions. We decomposed the optimal tax rate to two parts: a tax rate reflecting the focal pollutant and an additional tax component, the size of which depends on the cobenefits.

## Conclusion

We assessed in this paper how the costs of reducing emissions behave when cobenefits are taken into account. Also, we determined the implications of cobenefits in environmental policies. To this end, we derived the private costs of reducing nutrient runoff and GHG emissions using Finnish agricultural data. We then accounted for the cobenefits relevant for deriving the social costs of emissions reductions. Due to cobenefits, the social costs always lay below the private ones, indicating lower costs. GHG cobenefits from nutrient load reduction (and vice versa) create a gap between the privately and socially optimal abatement levels. The size of the gap depends on the valuation of the cobenefits. For a given marginal benefit function, the marginal cost of reducing the focus pollutant is lower with cobenefits than without cobenefits.

Our results suggests that accounting for multiple pollutants and their coeffects when applying different abatement measures is important, confirming similar findings of previous studies (e.g., Eory et al. 2018). The higher we value damage from pollution, the more attention we should give to these coeffects. The chosen damage values also directly affect the results for MCs with cobenefits in this study.

Our analysis has important implications for environmental policies. Accounting for cobenefits leads to a higher cap or tax on the pollutant on which the policy is focused. A uniform carbon tax levied on all agricultural production is extremely well-suited to policies focusing on GHG emissions, but it is impossible to levy an effluent tax directly on nutrient loading. For nutrient loading, a set of instruments would be needed: a nitrogen tax, a buffer strip subsidy and possible technology supports. The framework of marginal costs and benefits would only define the required abatement and would facilitate the comparison of marginal costs with dairy farms and other sectors.

For future research, the dairy management model could be modified for organic soils, and further measures, pollutants or effects could be added; additionally, marginal costs for reducing nutrient runoff could be separated for nitrogen and phosphorus runoff, as different measures may affect only one nutrient.

## Electronic supplementary material

Below is the link to the electronic supplementary material.

Supplementary material 1 (PDF 278 kb)
