# Supplementary material for: Multiple-pollutant cost-efficiency: Coherent water and climate policy for agriculture
Source: Ambio. 2019 Sep 24;48(11):1304–13. doi: 10.1007/s13280-019-01257-z (PMC6814639; doi:10.1007/s13280-019-01257-z)
Supplement: Supplementary file 1 — Supplementary material 1 (PDF 278 kb) [file 13280_2019_1257_MOESM1_ESM.pdf]

Ambio

Electronic Supplementary Material

This supplementary material has not been peer reviewed.

Title: **Multiple pollutant cost-efficiency: coherent water and climate policy for agriculture**

Authors: Sanna Lötjönen, Markku Ollikainen

ESM 1. Details of the studied measures

Dairy management

The dairy management model is based on Lötjönen et al. (unpubl. results) (see their article for additional details). The modeled farm consist of dairy cows for milk production and 10 separate field parcels at different distances (á 6 ha in 0.5 km intervals from the farm center with milk production facilities: [0.5, 1, 1.5, 2, 2.5, 3, 3.5, 4, 4.5, 5], i.e. 60 ha in total). Each parcel is cultivated with barley (sold to the market) or silage (used as feed within the farm) and fertilized with mineral fertilizer or manure (produced on farm). Manure can be exported outside the farm. Altering the shares of concentrate feeds and silage in the diet affects methane emissions from enteric fermentation, nutrient composition of manure and milk production. Manure is stored as slurry and the manure storage is either uncovered or covered with a floating cover. The cover prevents rain water from entering the storage and mixing with manure. Covering affects nitrogen volatilization as ammonia, and N_2O and CH_4 emissions. Nitrogen volatilization reduces manure nitrogen content, and thus more manure needs to be spread to achieve the same nitrogen fertilization. Herd size affects pasture area through stocking density. Total area of pasture land is 42 ha and any excess land is cultivated with barley. The model of Lötjönen et al. (unpubl. results) is slightly modified in the present calculations to gain matching nitrogen response function for barley, and nitrogen and phosphorus runoff functions, as are used in crop production measures. Also, we have updated parameter values to the year 2018. Thus, our results are not directly comparable with the original results in Lötjönen et al. (unpubl. results).

Crop production

Reducing mineral fertilization and increasing the share of buffer strips

Mineral N fertilization is reduced and buffer strip share of the field parcel is increased. Barley is used as a representative crop cultivated on different soil types (clay and loam combined as mineral soil, and organic soil) with mean soil quality and with different management practices (conventional tillage, no-till). Parameter values for nitrogen response functions are obtained from Ervola et al. (2018). With decreasing fertilization and increasing buffer strips yields are reduced causing losses to the farmer. Reduced fertilization results in declining nutrient runoff and GHG emissions from fertilizer manufacture and application, and increased buffer strips in decreased GHG emissions from cultivation and crop drying and reduced nutrient runoff.

Legumes in crop rotations

Barley monoculture with conventional tillage is replaced with crop rotations including legumes (red clover – grass) in mineral soils. For details of the crop rotation model, see Lötjönen and Ollikainen (2017). After applying legumes in crop rotations, GHG emissions and nutrient runoff can be further reduced by reducing mineral fertilization and increasing buffer strips. Compared to the study of Lötjönen and Ollikainen (2017), here economic parameter values are updated to the year 2018.

Catch crops

Catch crops are modelled based on Valkama et al. (2015). Non-legume catch crops are undersown simultaneously with barley. They capture excessive nitrogen in the soil and thus reduce N runoff by 50%, but also reduce grain yield by 3%. P runoff is not affected by assumption. According to Dalgaard et al. (2011), using catch crops increases soils carbon by 733 kg CO_2 ha-1 and reduces N_2O emissions by 185 kg CO_2e ha-1.

Afforestation

Organic or mineral soils in barley cultivation with conventional tillage are afforested. Private costs consist of lost profits from barley cultivation subtracted by discounted gains from future harvests. GHG emissions and nutrient runoff from cultivation are avoided. Calculations are based on Ervola et al. (2012).

Green fallow

Organic or mineral soils in barley cultivation with conventional tillage are transformed to green fallow with lower soil GHG emissions and nutrient runoff. GHG emissions relating to fertilization and cultivation practices are also avoided. Private costs include lost profits from barley cultivation. Calculations are based on Ervola et al. (2012).

Table S1. Estimated total area for different measures and number of dairy farms used in the aggregation (OSF 2018)

Measure	Estimated area 2018, ha	Share
Catch crop, mineral, conv	46 823	0.068
Crop rotation, mineral, conv.	165 821	0.241
Cereal mono, mineral, conv	284 820	0.414
Cereal mono, mineral, no-till	119 466	0.174
Cereal, organic, conv tillage	57 122	0.083
Cereal, orgainc, no-till	13 718	0.020
SUM	687 771	1
Dairy farms (assumed homogenous)		4583

ESM 2. Detailed result of MC calculations

Dairy management

Table S2. Optimal parameter values when reducing GHG emissions or nutrient runoff in dairy management (BL=baseline, i.e. 0% reduction)

	**GHG emission reduction**							
**Free** (BL 514 tCO_2e farm-1)	**0%**	**5%**	**10%**	**15%**	**20%**	**25%**	**30%**	**50%**
Herd size	61	56	51	45	39	34	29	18
Barley parcels (out of 10)	4	4	4	5	6	6	6	3
Concentrate intake (kg d-1)	17.5	17.0	16.6	17.2	17.9	17.3	16.8	12.8
Manure export (m^3 farm-1)	0	0	0	0	0	0	0	0
**CAP** (BL 549 tCO_2e farm-1)								
Herd size	70	64	58	52	47	42	36	26
Barley parcels (out of 10)	3	3	4	4	4	4	5	2
Concentrate intake (kg d-1)	17.5	16.6	17.1	16.4	16.0	15.8	16.2	14.2
Manure export (m^3 farm-1)	460	292	187	44	0	0	0	0
	**Nutrient runoff reduction**							
**Free** (BL 3233 kgNe farm-1)	**0%**	**5%**	**10%**	**15%**	**20%**	**25%**	**30%**	**50%**
Herd size	61	57	53	48	45	39	37	-
Barley parcels (out of 10)	4	4	4	4	3	3	1	-
Concentrate intake (kg d-1)	17.5	17.3	17.1	16.8	15.8	15.5	14.5	-
Manure export (m^3 farm-1)	0	0	0	0	0	0	0	-
**CAP** (BL 3296 kgNe farm-1)								
Herd size	70	64	62	57	53	49	42	-
Barley parcels (out of 10)	3	3	2	2	1	0	0	-
Concentrate intake (kg d-1)	17.5	17.1	16.1	15.9	15.2	14.7	14.6	-
Manure export (m^3 farm-1)	460	321	202	63	0	0	0	-

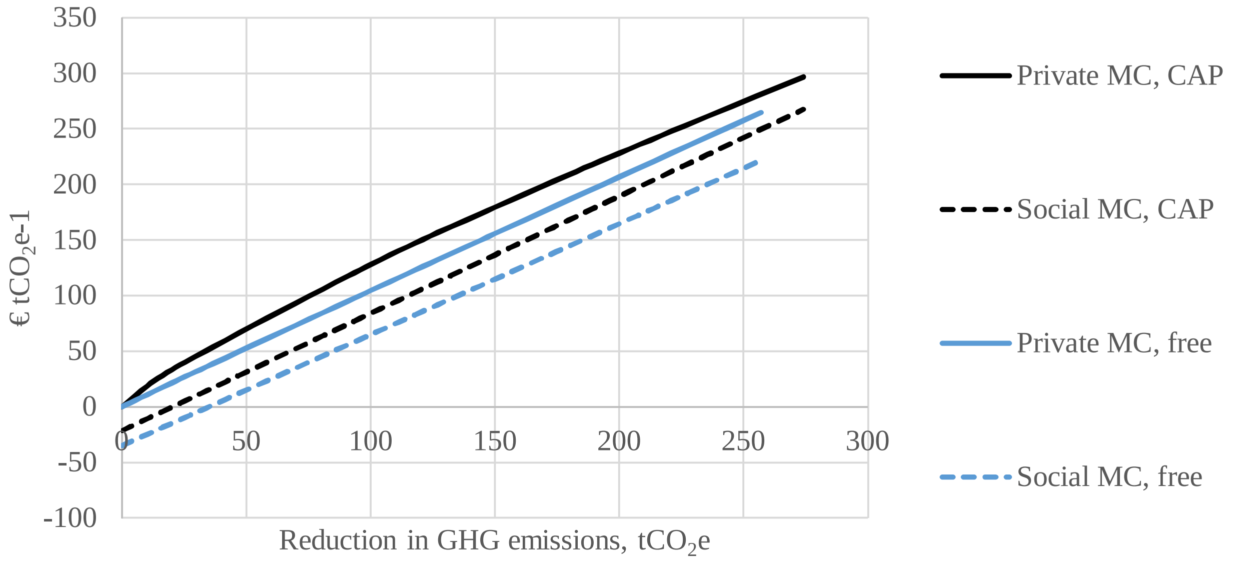

Figure S1. Marginal private and social cost of GHG emission reductions for dairy management under free market and CAP

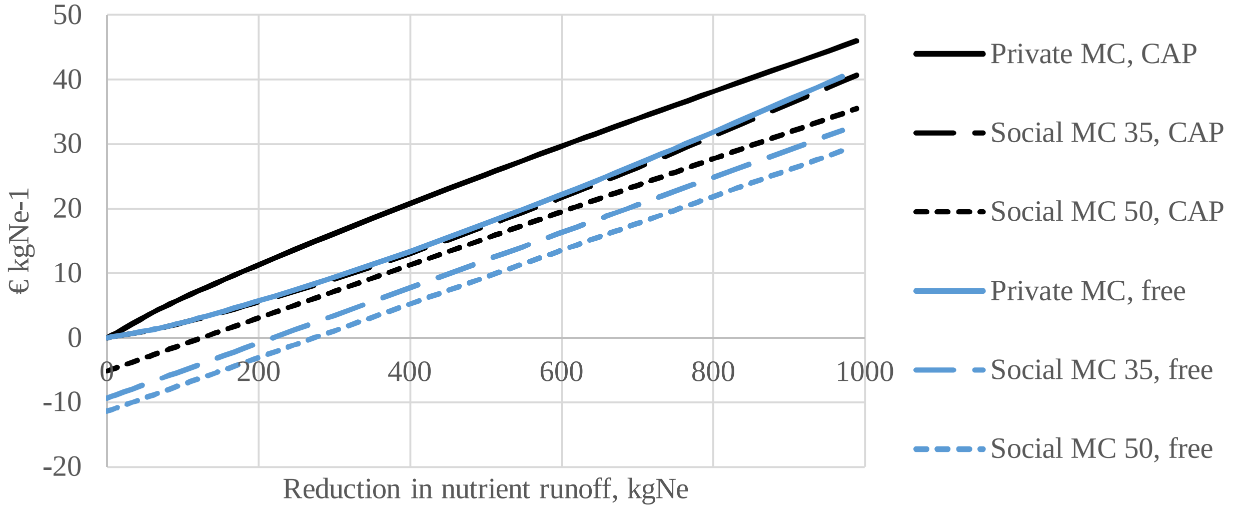

Figure S2. Marginal private and social cost of nutrient runoff reductions for dairy management under free market and CAP

Crop production

Table S3. Baseline GHG emissions and nutrient runoff for crop production under free market and CAP

Measure	GHG emissions tCO_2e ha-1	Nutrient runoff kgNe ha-1
Free market		
Crop, mineral, conv	2.5	26.6
Crop, mineral, no-till	3.0	18.8
Crop, organic, conv	16.1	51.1
Crop, organic, no-till	13.2	30.8
Catch crop, mineral, conv	1.6	16.9
Crop rotation, mineral, conv	1.7	20.8
CAP		
Crop, mineral, conv	2.3	15.3
Crop, mineral, no-till	2.8	13.0
Crop, organic, conv	15.7	28.0
Crop, organic, no-till	12.8	19.3
Catch crop, mineral, conv	1.4	10.5
Crop rotation, mineral, conv	1.6	13.1

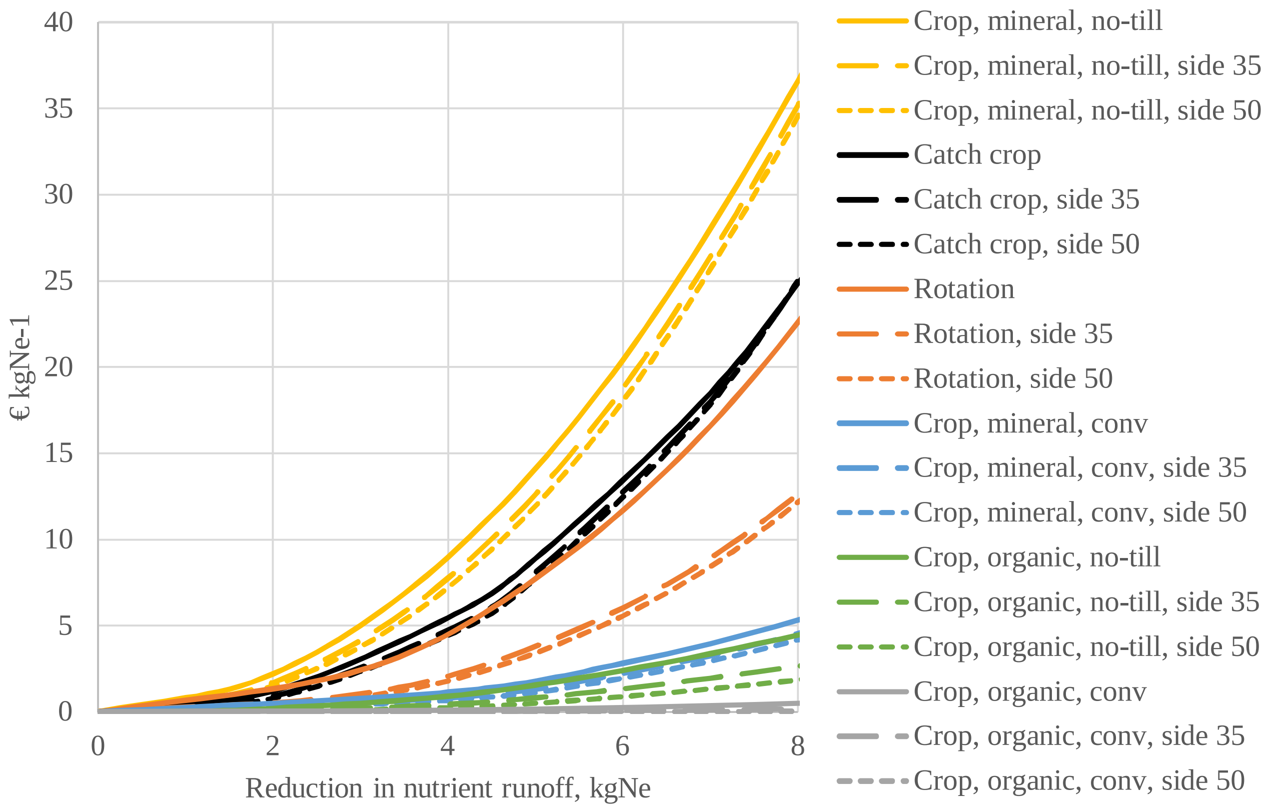

Figure S3. Private and social marginal costs of nutrient runoff reductions under different measures in crop production by reducing fertilization and increasing buffer strips under free market

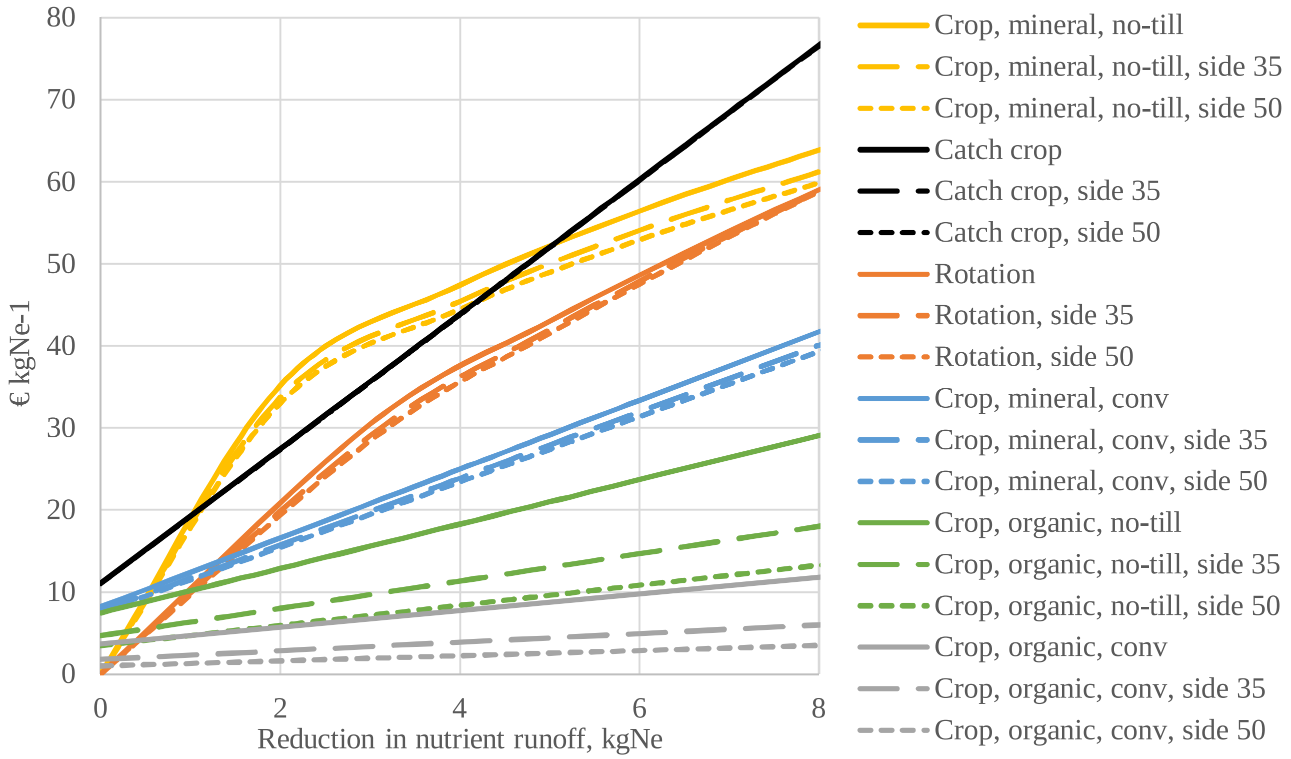

Figure S4. Private and social marginal costs of nutrient runoff reductions under different measures in crop production by reducing fertilization and increasing buffer strips under CAP

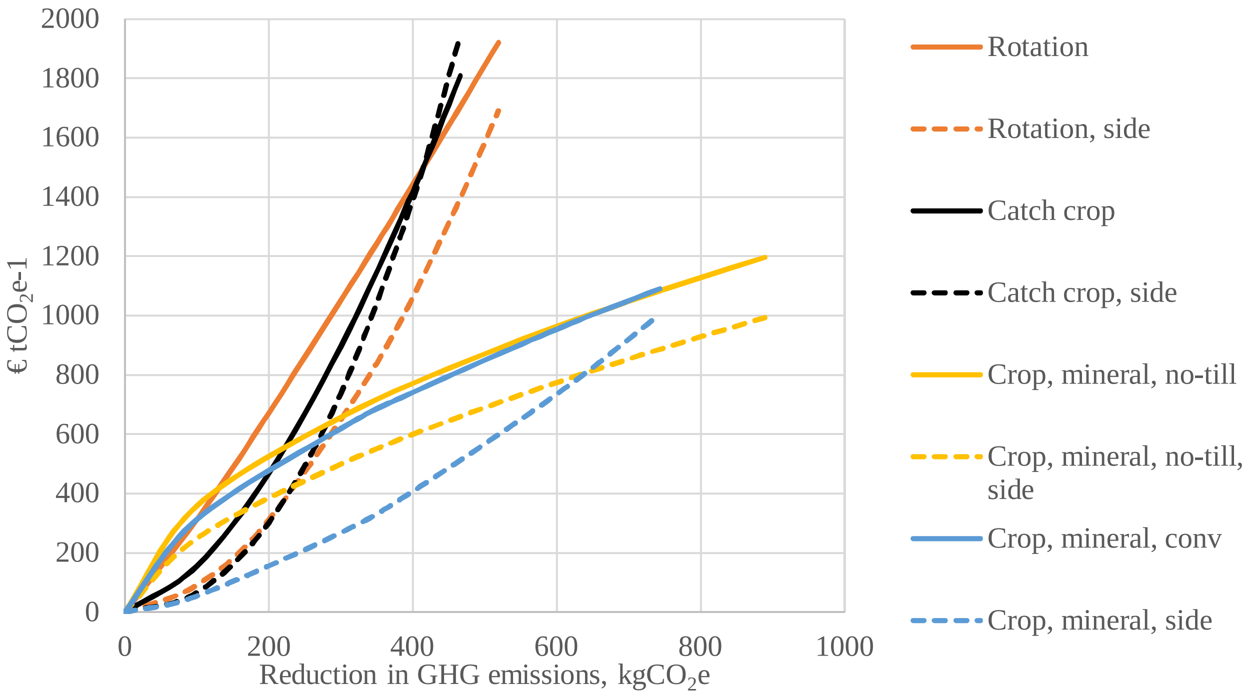

Figure S5. Private and social marginal costs of GHG emission reductions under different measures in crop production by reducing fertilization and increasing buffer strips in mineral soils under free market

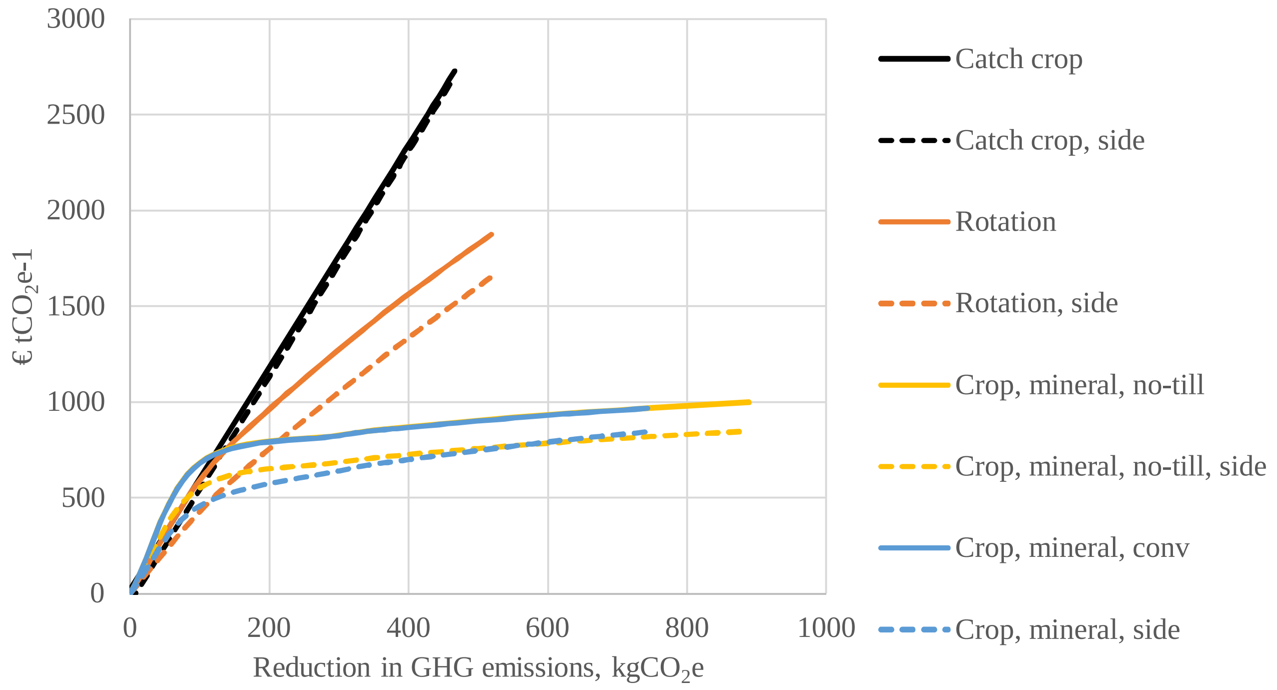

Figure S6. Private and social marginal costs of GHG emission reductions under different measures in crop production by reducing fertilization and increasing buffer strips in mineral soils under CAP

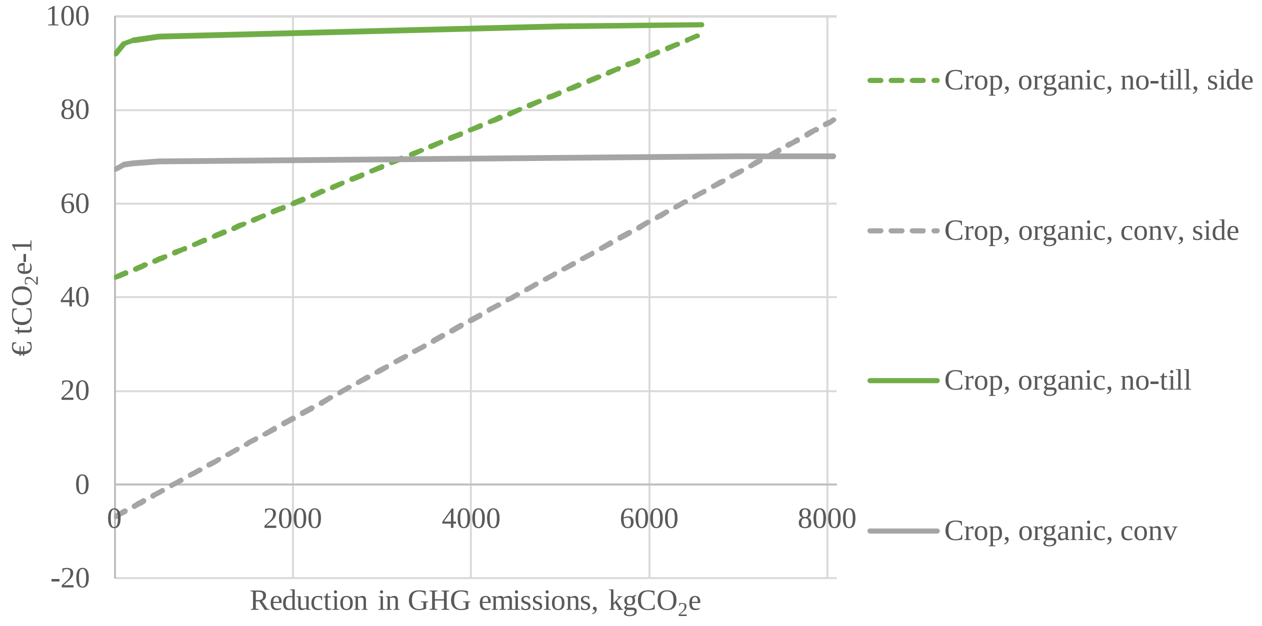

Figure S7. Private and social marginal costs of GHG emission reductions by reducing fertilization and increasing buffer strips with barley monoculture in organic soils under free market

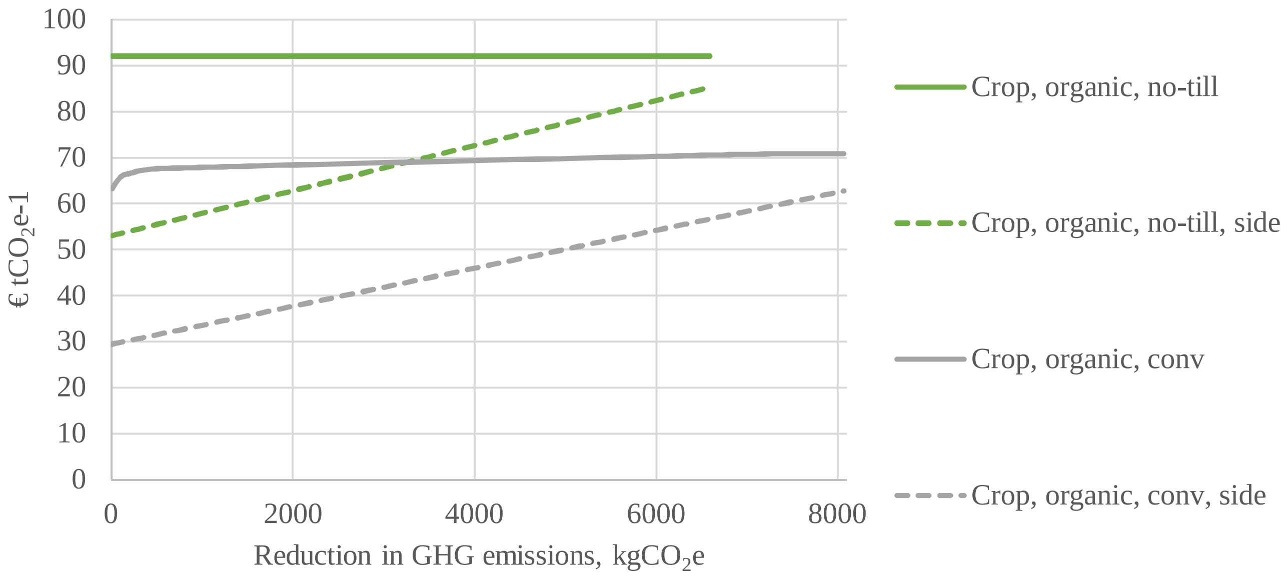

Figure S8. Private and social marginal costs of GHG emission reductions by reducing fertilization and increasing buffer strips with barley monoculture in organic soils under CAP

Table S4. Nitrogen fertilization (kg N ha-1) when reducing nutrient runoff under free market and CAP

	**Reduction in nutrient runoff**							
**Measure**	**0%**	**5%**	**10%**	**15%**	**20%**	**25%**	**30%**	**50%**
Free market								
Crop, mineral, conv	134	134	133	130	127	124	120	107
Crop, mineral, no-till	132	131	128	125	120	116	112	106
Crop, organic, conv	131	131	129	127	124	121	118	104
Crop, organic, no-till	129	128	126	123	120	116	112	99
Catch crop, mineral, conv	133	132	131	129	126	123	120	111
Crop rotation, mineral, conv	85	84	83	80	77	73	69	54
CAP								
Crop, mineral, conv	100	100	100	100	100	100	100	100
Crop, mineral, no-till	100	100	100	100	100	100	100	100
Crop, organic, conv	100	100	100	100	100	99	98	97
Crop, organic, no-till	100	100	100	100	100	100	100	100
Catch crop, mineral, conv	100	100	100	100	100	100	100	100
Crop rotation, mineral, conv	70	51	31	18	18	18	18	-

Table S5. Nitrogen fertilization (kg N ha-1) when reducing GHG emissions under free market and CAP

	Reduction in GHG emissions							
**Measure**	**0%**	**5%**	**10%**	**15%**	**20%**	**25%**	**30%**	**50%**
Free market								
Crop, mineral, conv	134	106	77	64	64	64	64	-
Crop, mineral, no-till	132	98	63	57	57	57	57	-
Crop, organic, conv	131	121	121	121	121	121	121	121
Crop, organic, no-till	129	115	115	115	115	115	115	115
Catch crop, mineral, conv	133	115	97	79	61	43	25	-
Crop rotation, mineral, conv	85	65	45	25	18	18	18	-
CAP								
Crop, mineral, conv	100	73	64	64	64	64	64	-
Crop, mineral, no-till	100	67	60	60	60	60	60	-
Crop, organic, conv	100	100	100	100	100	100	100	100
Crop, organic, no-till	100	100	100	100	100	100	100	100
Catch crop, mineral, conv	100	83	66	50	33	16	-	-
Crop rotation, mineral, conv	70	59	58	56	54	53	52	50

Table S6. Buffer strip width (m) when reducing nutrient runoff under free market and CAP

	Reduction in nutrient runoff							
**Measure**	**0%**	**5%**	**10%**	**15%**	**20%**	**25%**	**30%**	**50%**
Free market								
Crop, mineral, conv	0.0	0.0	0.0	0.2	0.4	0.7	1.3	7.2
Crop, mineral, no-till	0.0	0.0	0.1	0.4	1.0	1.9	3.5	18.9
Crop, organic, conv	0.0	0.0	0.0	0.1	0.3	0.6	1.0	5.5
Crop, organic, no-till	0.0	0.0	0.0	0.2	0.5	1.0	1.8	10.4
Catch crop, mineral, conv	0.0	0.0	0.0	0.2	0.5	0.1	2.0	10.7
Crop rotation, mineral, conv	0.0	0.0	0.0	0.2	0.4	0.7	1.3	7.7
CAP								
Crop, mineral, conv	3.0	4.1	5.5	7.1	9.0	11.1	13.6	27.3
Crop, mineral, no-till	3.0	4.7	6.9	9.6	12.7	16.3	20.2	39.4
Crop, organic, conv	3.0	4.0	5.2	6.6	8.3	10.1	12.0	23.7
Crop, organic, no-till	3.0	4.3	6.0	7.9	10.3	12.9	16.0	32.3
Catch crop, mineral, conv	3.0	4.2	5.8	7.6	9.8	12.3	15.2	30.7
Crop rotation, mineral, conv	3.0	3.0	3.0	9.0	25.7	42.5	59.3	-

Table S7. Buffer strip width (m) when reducing GHG emissions under free market and CAP

	Reduction in GHG emissions							
**Measure**	**0%**	**5%**	**10%**	**15%**	**20%**	**25%**	**30%**	**50%**
Free market								
Crop, mineral, conv	0.0	0.0	0.0	8.5	24.5	40.4	56.4	-
Crop, mineral, no-till	0.0	0.0	0.0	17.7	39.3	60.8	82.4	-
Crop, organic, conv	0.0	7.3	15.1	22.8	30.5	38.3	46.0	77.0
Crop, organic, no-till	0.0	8.1	16.9	25.7	34.6	43.4	52.3	87.6
Catch crop, mineral, conv	0.0	0.0	0.0	0.0	0.0	0.0	0.0	-
Crop rotation, mineral, conv	0.0	0.0	0.0	0.0	11.9	29.5	47.1	-
CAP								
Crop, mineral, conv	3.0	3.0	13.1	27.9	42.7	57.5	72.3	-
Crop, mineral, no-till	3.0	3.0	19.0	39.1	59.1	79.1	99.2	-
Crop, organic, conv	3.0	10.6	18.2	25.8	33.4	41.0	48.6	78.9
Crop, organic, no-till	3.0	11.7	20.4	29.1	37.8	46.4	55.1	89.9
Catch crop, mineral, conv	3.0	3.0	3.0	3.0	3.0	3.0	-	-
Crop rotation, mineral, conv	3.0	3.2	4.4	5.8	7.5	9.5	11.9	26.4

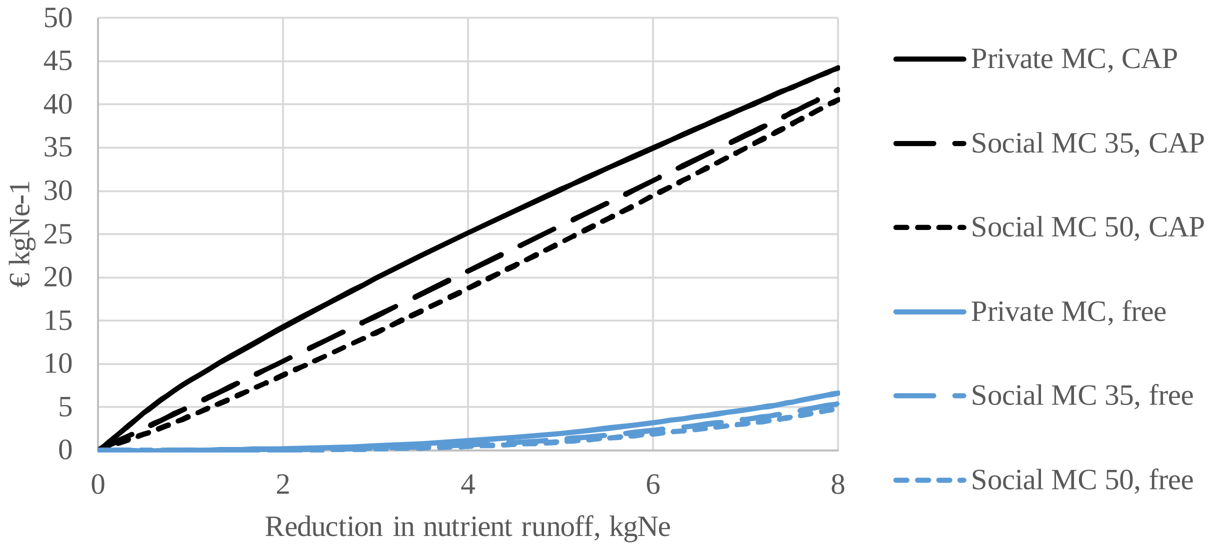

Figure S9. Aggregated private and social marginal cost curves for reducing nutrient runoff in crop production

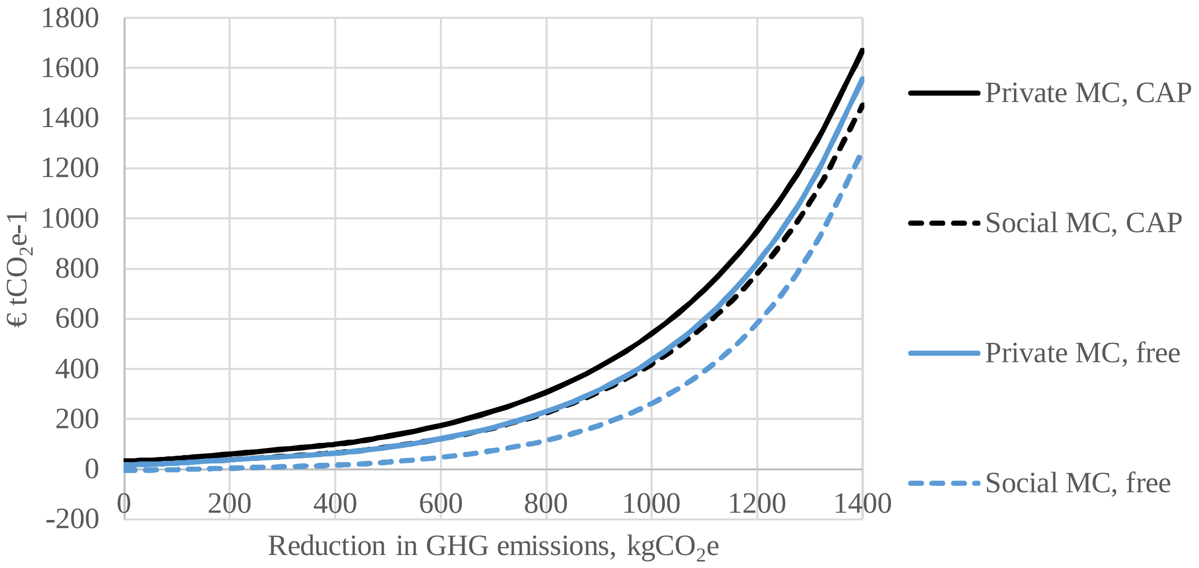

Figure S10. Aggregated private and social marginal abatement cost curves for reducing GHG emissions in crop production

Table S8. Nutrient runoff abatement for individual measures (kgNe ha-1) with alternative total abatement levels in the aggregated private marginal cost functions for crop production

**Measure**	**Total abatement, kgNe ha-1**		
Free	**1**	**2**	**5**
Crop, mineral, conv	1.07	2.14	5.32
Crop, mineral, no-till	0.34	0.73	1.98
Crop, organic, conv	3.10	6.22	15.61
Crop, organic, no-till	1.16	2.32	5.82
Catch crop, mineral, conv	0.51	1.03	2.61
Crop rotation, mineral, conv	0.75	1.47	3.57
CAP			
Crop, mineral, conv	0.57	1.63	4.65
Crop, mineral, no-till	0.12	0.28	1.15
Crop, organic, conv	6.83	11.21	23.64
Crop, organic, no-till	1.16	2.81	7.47
Catch crop, mineral, conv	-	0.49	2.03
Crop rotation, mineral, conv	0.63	1.06	2.60

Table S9. GHG emission abatement for individual measures ($kgCO_2e$ ha-1) with alternative total abatement levels in the aggregated private marginal cost functions for crop production

Measure	Total abatement, $kgCO_2e$ ha-1		
Free	**500**	**1000**	**1400**
Crop, mineral, conv	9	252	743
Crop, mineral, no-till	5	221	889
Crop, organic, conv	5843	8068	8068
Crop, organic, no-till	-	6589	6589
Catch crop, mineral, conv	61	223	434
Crop rotation, mineral, conv	26	169	443
CAP			
Crop, mineral, conv	-	260	743
Crop, mineral, no-till	-	254	889
Crop, organic, conv	6003	8068	8068
Crop, organic, no-till	-	6589	6589
Catch crop, mineral, conv	8	135	302
Crop rotation, mineral, conv	5	155	480

Table S10. Results for reducing nutrient runoff with "technological choices"

Free	**Baseline* (kgNe ha-1)**	**Reduced runoff (kg ha-1)**	**Private cost** (€ ha-1)**	**Private marginal cost (€ kgNe-1)**	**Social marginal cost (35) (€ kgNe-1)**	**Social marginal cost (50) (€ kgNe-1)**
Afforestation, mineral	25.1	22.4	346	15.5	3.8	-1.1
Afforestation, organic	26.3	23.7	294	12.4	-6.4	-14.4
Green fallow, mineral	25.1	13.6	428	31.5	24.7	21.7
Green fallow, organic	26.3	14.9	376	25.3	0.8	-9.7
CAP						
Afforestation, mineral	2.3	12.9	846	25.7	6.0	-2.5
Afforestation, organic	15.5	10.5	724	19.9	-20.7	-38.1
Green fallow, mineral	2.3	4.0	928	102.3	80.7	71.5
Green fallow, organic	15.5	1.6	806	177.9	-33.2	-123.7

*Barley cultivation with conventional tillage is used as baseline.

**Private cost includes the lost area-based subsidy (not included in the social cost).

Table S11. Results for reducing GHG emissions with "technological choices"

Free	Baseline* (tCO_2e ha-1)	Reduced GHG (t ha-1)	Private cost** (€ ha-1)	Private marginal cost (€ tCO_2e-1)	Social marginal cost (€ tCO_2e-1)
Afforestation, mineral	2.4	7.4	346	46.6	19.4
Afforestation, organic	16.1	12.7	294	23.2	6.4
Green fallow, mineral	2.4	2.7	428	161.0	115.0
Green fallow, organic	16.1	10.4	376	36.1	23.3
CAP					
Afforestation, mineral	2.3	7.3	846	45.6	29.7
Afforestation, organic	15.5	12.1	724	17.2	9.4
Green fallow, mineral	2.3	2.5	928	165.9	151.3
Green fallow, organic	15.5	9.9	806	29.5	28.0

*Barley cultivation with conventional tillage is used as baseline.
**Private cost includes the lost area-based subsidy (not included in the social cost).

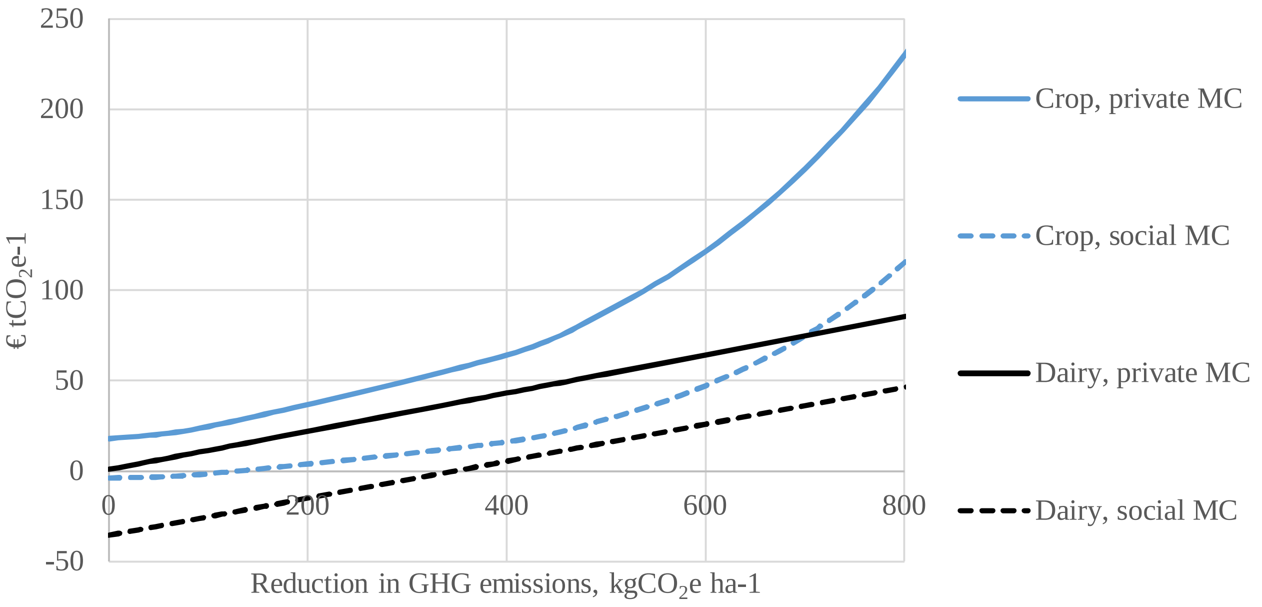

Figure S11. Illustration of aggregated hectare-based marginal cost curves for GHG emission reductions in crop production with measures allowing for cultivation and in dairy management (note: hectare-based curve for dairy management is arbitrary due to varying field area)

Crop production and dairy management combined

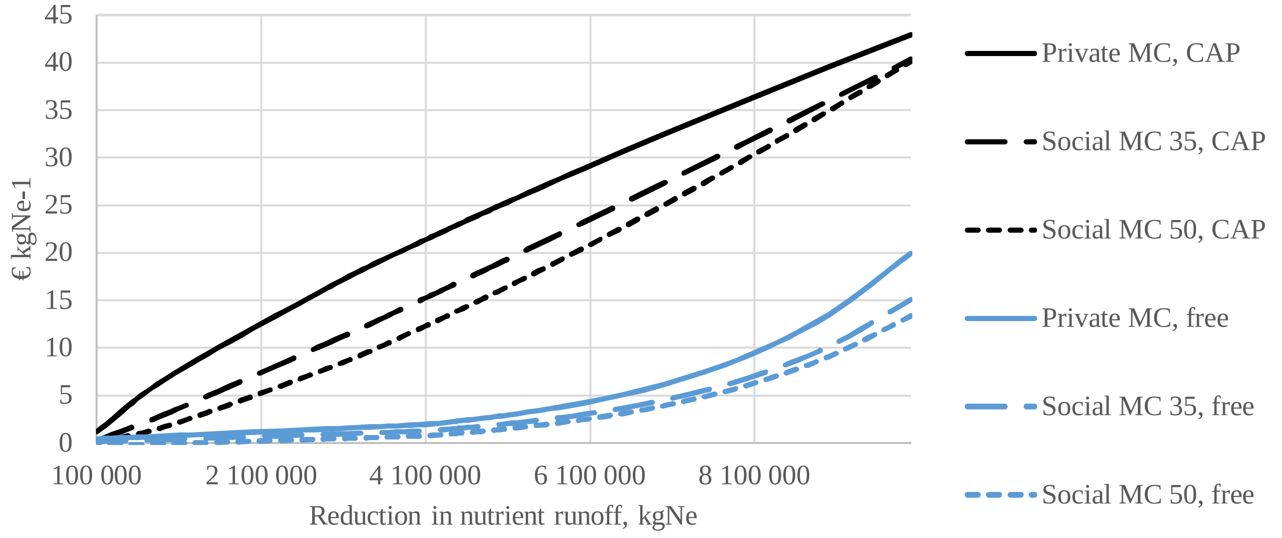

Figure S12. Aggregated private and social marginal cost curves for reducing nutrient runoff in crop production and dairy management combined under free market and CAP

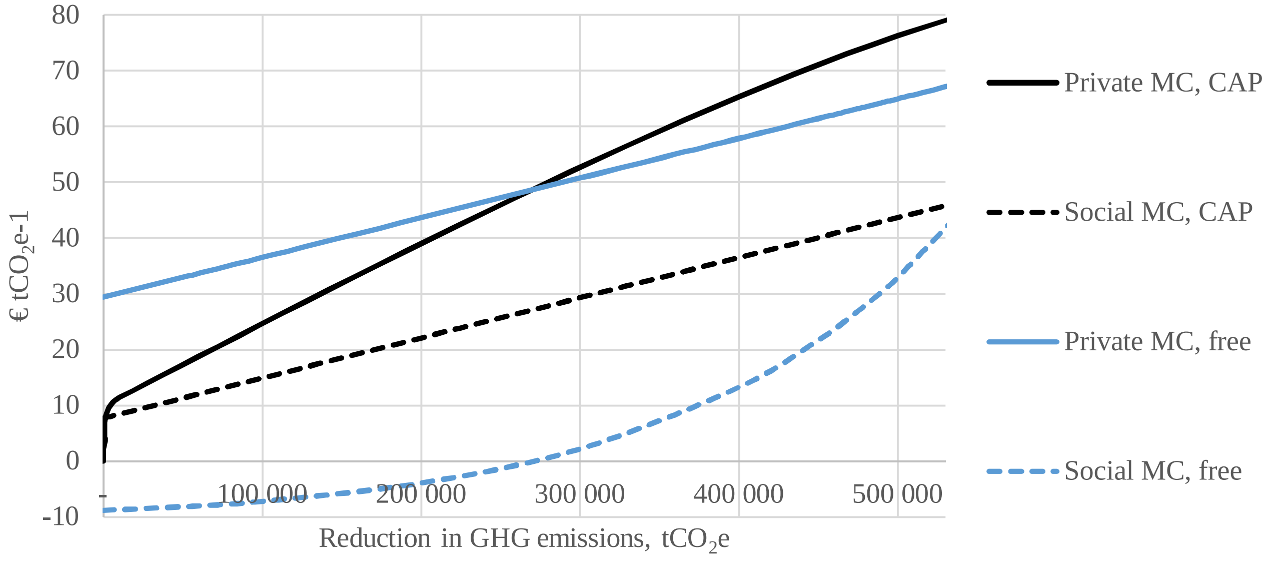

Figure S13. Aggregated private and social marginal cost curves for reducing GHG emissions in crop production and dairy management combined under free market and CAP

Table S12. Nutrient runoff and GHG emission reductions for individual measures with a chosen total abatement level in the aggregated private marginal cost functions for crop production and dairy management combined (nutrient runoff reduction is per hectare for crop production measures and per dairy farm for dairy management)

**Measure**	**Nutrient runoff reduction, kgNe**	**GHG emissions reduction, kgCO_2e**
**Free**	**687 770**	**343 885 000**
Crop, mineral, conv	1.04	9
Crop, mineral, no-till	0.33	5
Crop, organic, conv	3.00	559
Crop, organic, no-till	1.12	-
Catch crop, mineral, conv	0.50	60
Crop rotation, mineral, conv	0.73	25
Dairy management	5.04	65 838

Table S13. Total cost functions for reducing GHG emissions and nutrient runoff under free market

**Aggregated for crop production**	
Nutrient stand-alone	$0.00885x^{3.571}$
Nutrient with GHG co-benefit 35	$0.002343x^{4.052}$
Nutrient with GHG co-benefit 50	$0.001056x^{4.347}$
GHG stand-alone	$5.619e^{0.003189x}$
GHG with nutrient co-benefit	$-23.56e^{0.0004174x} + 1.562e^{0.003836x}$
**Aggregated for dairy management**	
Nutrient stand-alone	$0.02095x^{4.223}$
Nutrient with GHG co-benefit 35	$0.005801x^{4.671}$
Nutrient with GHG co-benefit 50	$0.00208x^{5.049}$
GHG stand-alone	$0.04664x^{2.292}$
GHG with nutrient co-benefit	$-271.9 - 103.4x - 0.02208x^2 + 0.0001893x^3$
**Jointly aggregated for crop and dairy**	
Nutrient stand-alone	$1020000.0e^{3.911\times10^{-7}x}$
Nutrient with GHG co-benefit 35	$-3578000.0e^{6.109\times10^{-8}x} + 827500.e^{3.8769999999999999\times10^{-7}x}$
Nutrient with GHG co-benefit 50	$-7655000.0e^{1.7969999999999999\times10^{-7}x} + 3506000.0e^{3.0179999999999999\times10^{-7}x}$
GHG stand-alone	$-1249000.0 + 0.02941x + 3.556\times10^{-11}x^2$
GHG with nutrient co-benefit	$-2.286\times10^{7}e^{5.2199999999999999\times10^{-10}x} + 568500.e^{5.484\times10^{-9}x}$

Table S14. Total cost functions for reducing GHG emissions and nutrient runoff under CAP

**Aggregated for crop production**	
Nutrient stand-alone	$4.475x^{1.815}$
Nutrient with GHG co-benefit 35	$2.558x^{2.007}$
Nutrient with GHG co-benefit 50	$1.892x^{2.114}$
GHG stand-alone	$11.43e^{0.00282x}$
GHG with nutrient co-benefit	$6.054e^{0.003105x}$
**Aggregated for dairy management**	
Nutrient stand-alone	$6.635x^{2.662}$
Nutrient with GHG co-benefit 35	$4.307x^{2.781}$
Nutrient with GHG co-benefit 50	$2.495x^{2.975}$
GHG stand-alone	$0.1436x^{2.148}$
GHG with nutrient co-benefit	$0.005162x^{2.557}$
**Jointly aggregated for crop and dairy**	
Nutrient stand-alone	$0.00008087x^{1.782}$
Nutrient with GHG co-benefit 35	$4.452 \times 10^{-7}x^{2.091}$
Nutrient with GHG co-benefit 50	$9.168 \times 10^{-9}x^{2.325}$
GHG stand-alone	$0.000003101x^{1.485}$
GHG with nutrient co-benefit	$-4475000.0 + 0.007717x + 3.596 \times 10^{-11}x^2$

References

Dalgaard T., J.E. Olesen, S.O. Petersen, B.M. Petersen, U. Jørgensen, T. Kristensen, N.J. Hutchings, S. Gyldenkærne and J.E. Hermansen. 2011. Developments in greenhouse gas emissions and net energy use in Danish agriculture – How to achieve substantial CO2 reductions? *Environmental Pollution* 159:3193-3203.

Ervola A., J. Lankoski and M. Ollikainen. 2018. Climate and Water Quality Policy Design for Agriculture with Environmental Co-Benefits. *Modern Concepts & Developments in Agronomy* 3(1).

Ervola A., J. Lankoski, M. Ollikainen and H.J. Mikkola. 2012. Agriculture and climate change: The socially optimal production, land use, and GHG emissions. *Food Economics* 9:10-24.

Lötjönen S. and M. Ollikainen. 2017. Does crop rotation with legumes provide an efficient means to reduce nutrient loads and GHG emissions? *Review of Agricultural, Food and Environmental Studies* 98:283-312.

OSF. 2018. Official Statistics of Finland, Natural Resources Institute Finland, Utilized agricultural area 2018.

Valkama E., R. Lemola, H. Känkänen and E. Turtola. 2015. Meta-analysis of the effects of undersown catch crops on nitrogen leaching loss and grain yields in the Nordic countries. *Agriculture, Ecosystems & Environment* 203:93-101.
